# Behavioral and Morphological Effects of Natural Sweeteners on *Caenorhabditis elegans*

**DOI:** 10.17912/micropub.biology.002130

**Published:** 2026-05-22

**Authors:** Diya Patel, Crystal Shults, Jacob. R Manjarrez

**Affiliations:** 1 Biochemistry and Microbiology, Oklahoma State University Center for Health Sciences, Tulsa, OK, US

## Abstract

*Caenorhabditis elegans* is an attractive, genetically tractable model organism widely used to investigate morphological and locomotor responses to environmental and dietary factors. This study examined the effects of the natural alternative sweeteners, Stevia in the Raw®, Truvia®, Lakanto®, and Monkfruit in the Raw®. Worms were exposed to each sweetener through their growth media, and behavioral (crawling and swimming speed, wavelength, and activity) and morphological (length, width, and area) parameters were quantified using WormLab.&nbsp; The objective was to evaluate the effects on development and behavior using natural alternative sweeteners relative to conventional granulated white sugar when administered in equivalent amounts.

**Figure 1. Morphological and Locomotor Analysis:** WormLab (MBF) software was used to analyze accumulated *C. elegans* data.&nbsp;&nbsp; **A)** Only crawling data with an algorithmic fit > 0.9 were included to ensure accuracy, which quantifies how well the worm model conforms to the underlying image data. The higher the number, the better the fit. The value 1.0 represents a perfect fit. GraphPad was used to create the box and whisker plots displaying the min and max of the sample.&nbsp; Significance is associated with the listed P value. Morphological metrics **B)** Length, **C)** Width, and **D)** Area were calculated using WormLab.&nbsp; Crawling metrics **E)** Wavelength and **F)** Center point speed (to remove extraneous head movement) were captured from recorded videos and calculated using WormLab.&nbsp; **Table** : Listed cumulative (n = animals) for crawling, swimming, and fit analysis based on two biological repeats.&nbsp; **G** ) Swimming metrics (cyan boxed), Activity (Represents the brush stroke (Refers to the area "painted" by the animal's body in a single complete stroke) normalized by the time taken to perform the two strokes, and **H)** Swim Speed (Refers to the traveling swimming speed of an animal measured over a two-stroke interval). **I)** Categorical map (dark blue boxed) of changing conditions set relative to GWS to show variation in treatment throughout the morphological and locomotor metrics.&nbsp; All experimental evidence was shown using 1-day-old adults based on two biological repeats.&nbsp; Significance based on Nested t-test (GraphPad, Dotmatics, version 11), P< 0.05.&nbsp; Granulated white sugar (GWS), Truvia® Natural Sweetener (TS), Lakanto® Monkfruit Sweetener (MS), Stevia in the Raw® (SR), or Monkfruit in the Raw® (MR).&nbsp;

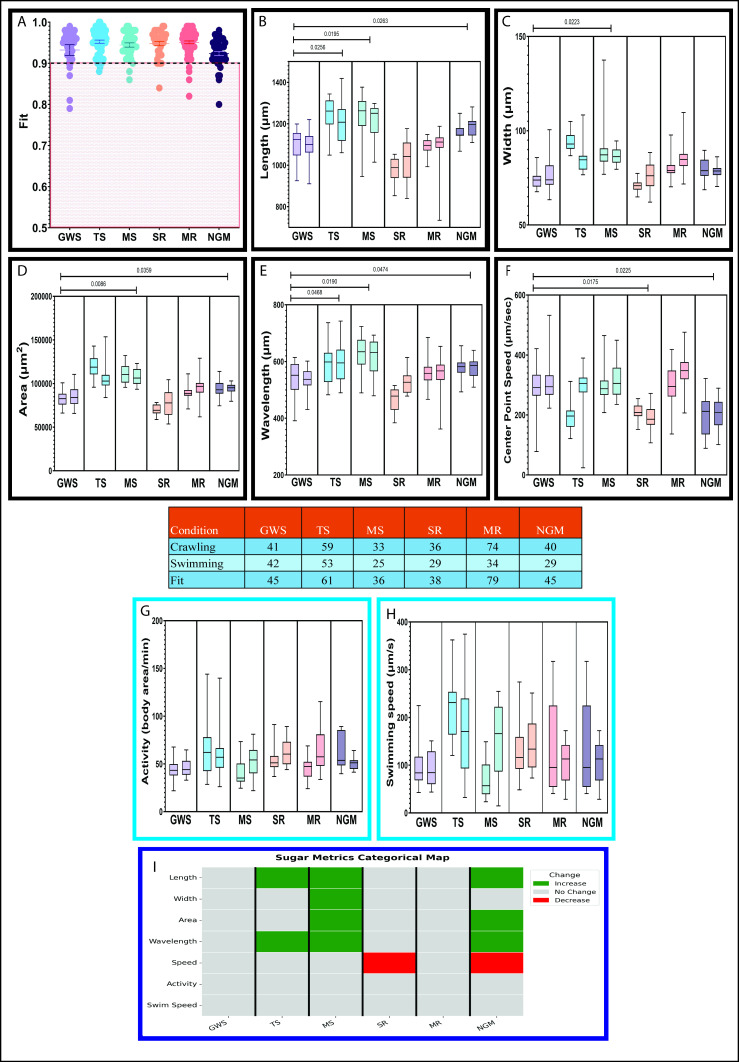



## Description

*C. elegans* is a well‑established model organism for studying development, neurobiology, and metabolism due to its short life cycle, genetic tractability, and well‑characterized physiology (Riddle 1997). In this study, we investigated the physiological and behavioral responses of *C. elegans* to commercially available household natural sweeteners containing dextrose, erythritol, or maltodextrin.

Erythritol, a common component of Lakanto® Monkfruit Sweetener and Truvia® Natural Sweetener, has been associated with increased cardiovascular risk and, more recently, a heightened likelihood of thrombotic events leading to stroke (Wein, 2025; Berry et al., 2025). Maltodextrin, present in Monkfruit in the Raw®, has been implicated in intestinal inflammation and metabolic dysregulation (Arnold & Chassaing, 2019). Dextrose, found in Stevia in the Raw®, has been linked to reduced cardiovascular endurance (Axflord, 1984). In contrast, the primary sweetening agents in these products, steviol glycosides and monk fruit mogrosides, are non‑nutritive compounds that do not significantly elevate blood glucose levels, contributing to their widespread use among individuals with diabetes or weight‑management concerns (Wazir et al., 2025; Satish et al., 2025; Yeung, 2023; Kaim & Labus, 2025; Muñoz-Labrador et al., 2023). Consistent with these compositional differences, with specific sweetener formulations, there have been observed differential effects likely arising from the chemical composition of the additives, despite steviol glycosides and mogrosides exhibiting sweetness intensities approximately 150–300 times greater than that of sucrose (Wazir et al., 2025; Satish et al., 2025; Dragomir et al., 2025). Both sweeteners have achieved Generally Recognized as Safe (GRAS) status in the United States and are widely incorporated into foods and beverages (Satish et al., 2025; Yeung, 2023). However, monk fruit remains only partially approved in the European Union, pending additional toxicological evaluation, a regulatory trajectory similar to that previously observed for stevia (Yeung, 2023; Kaim & Labus, 2025).

This study analyzed the average granulated white sugar (GWS) equivalent in a soft drink (13 – 8 tsp) (The Nutrition Source, 2009, www.hsph.harvard.edu/nutritionsource/healthy-drinks/) compared with equivalent quantities of Truvia® Natural Sweetener (TS), Lakanto® Monkfruit Sweetener (MS), Stevia in the Raw® (SR), and Monkfruit in the Raw® (MR), this is based on &nbsp;2 tsp equal to one packet association (for most sweeteners). NGM was used as a non‑sugar control. This experimental design was employed to assess the effects of natural alternative sweeteners on morphology and locomotor performance. Animals were raised on the designated sugar compositions (see Methods) from birth to the experimental age of one-day-old adults. The objective was to determine whether natural alternative sweeteners conferred physiological and behavioral effects over conventional granulated white sugar independent of calories, reflected by visible morphological outcomes (length, width, and area associated with growth) and locomotor metrics, including crawling speed and wavelength, as well as swimming activity and swimming speed.

We observed specific sweetener effects on growth and behavior. TS and MS promoted increased growth, with MS worms showing enlargement across all measured growth metrics ( Figure 1B- D & I). TS and MS worms exhibited longer wavelengths without a change in locomotor speed ( Figure 1E & I).&nbsp; &nbsp;SR worms showed slower crawling speeds similar to the no‑sugar NGM control ( Figure 1F & I). However, supplementation with natural alternative sweeteners did not produce significant changes in swimming behavior ( Figure 1G & H).

Erythritol-containing sweeteners (TS, MS) promoted increased growth in *C. elegans* . TS and MS produced showed a wavelength change during crawling. Inclusion of maltodextrin in MS seems to result in increased body length, width, and area, accompanied by increased locomotor wavelength. In contrast, the dextrose-containing formulation of SR reduced crawling speed without any growth-specific changes.&nbsp; The maltodextrin-containing MR did not show any changes relative to GWS.&nbsp; Collectively, these findings indicate that natural sweeteners can exert distinct effects on organismal physiology and behavior, with potential future implications on metabolic health.

Together, these findings demonstrate that sweetener composition can influence both growth and locomotor outcomes in *C. elegans* , highlighting the potential for physiological and behavioral effects of commonly used sugar substitutes.

Notably, Erythritol-containing sweeteners consistently increased morphological measures regardless of the alternative sweetener used, including alteration of a key locomotion variable, wavelength ( Figure 1B- E). In contrast, stevia-based sweeteners exhibited additive-dependent locomotor response, producing either a decrease or no change in locomotor speed ( Figure 1F ). These results suggest that the physiological and locomotor effects of commonly used natural alternative sweeteners have a dependence on the specific additive used in their formulations.

Many neurotoxic or neurodevelopmental insults alter locomotor speed or frequency; however, wavelength is typically more constrained (Karbowski et al., 2006; Fang-Yen et al., 2010; Butler et al., 2015); thus, deviations in wavelength may be particularly informative indicators of neural dysfunction (Abdelhack, 2022; Long et al., 2023; Petratou et al., 2024). Under normal conditions, wavelength remains relatively constant and is largely independent of frequency or developmental stage (Karbowski et al., 2006; Fang-Yen et al., 2010; Butler et al., 2015). The observed variation in locomotor wavelength following supplementation of different sugars may reflect altered signaling within the motor circuits governing *C. elegans* locomotion (Karbowski et al., 2006; Fang-Yen et al., 2010; Butler et al., 2015).

The consistent deviations observed in the erythritol-containing sweeteners may be indicative of neurophysiological impairment arising either from the natural sugar substitutes themselves or from metabolic by-products generated through interactions with the bacterial food source. This finding is of particular interest given recent associations between erythritol consumption and an increased risk of blood clot events and stroke in humans (Wein, 2025; Berry et al., 2025).

Consistent with the compositional differences, our findings show that growth and locomotor outcomes vary with the specific alternative sweetener.&nbsp; Among monk fruit and stevia sweeteners, the observed differential effects appear to arise from differences in the chemical composition of their additives.

## Methods

*C. elegans* strain and maintenance: The N2 strain (CGC; University of Minnesota) was maintained on nematode growth medium (NGM), in 60mm petri plates seeded with *Escherichia coli* strain OP50 , at 20°C.&nbsp; Media was supplemented with one of the following sweeteners:- Stevia in the Raw® (SR; stevia +dextrose)- Truvia® Natural Sweetener (TS; stevia + erythritol)- Lakanto® Monkfruit Sweetener (MS; monk fruit + erythritol)- Monk fruit in the Raw® (MR; monk fruit + maltodextrin)- Granulated white sugar (GWS; control)

Preparations of treatment plates: NGM agar plates with granulated white sugar (GWS, 1 tsp=4.2g) or sugar substitute were prepared equivalent to .084g/ml (GWS).&nbsp; Stevia in the Raw (Stevia and Maltodextrin, 1 packet = 2 tsp sugar) (SR), Truvia Natural Sweetener (TS, ¾ tsp = 2 tsp sugar), Lakanto Monk fruit Sweetener (MS, 1 packet = 2 tsp sugar), and Monk fruit in the Raw (MR, 1 packet=2 tsp GWS) were used. Plates were seeded with *Escherichia coli* strain OP50 .

Experiment design: The N2 strain was grown on the GWS, TS, MS, SR, MR, and NGM for three generations before synchronization. Synchronous L4 populations were achieved by gravid adult worms placed on NGM plates containing GWS, TS, MS, SR, MR, and NGM for a timed egg lay, and, after two hours, were removed, and synchronized eggs were allowed to develop at 20°C.

Video recording:&nbsp; Videos were recorded using a Leica SApo dissecting scope with a Leica Flexcam c5 at 30 fps.&nbsp; For crawling assays, worms were transferred to plates without OP50 for tracking.&nbsp; The worms were transferred from the growth plate into a 10 µL drop of water on the tracking plate.&nbsp; They were allowed to equilibrate for five min before recording for one min.&nbsp; For the swimming assay, the worms were transferred to a 20 µL drop of M9 on a microscope slide and recorded for 30 seconds. Data Analysis and Visualization: WormLab software was used to quantify crawling behavior (wavelength and center-point speed), swimming behavior (speed and activity), and morphological parameters (length, width, and area). Only data with a fit value > 0.9 were included in the crawling analysis. Statistical significance was determined using a nested t-test (GraphPad, Dotmatics, version 11), with p < 0.05 considered significant.

Phenotypic metric changes:&nbsp; Were categorized as increase, decrease, or no change relative to a reference condition and recorded for each strain-condition combination at day 1 of adulthood (1 DOA). Data were organized across seven morphological and locomotor metrics (length, width, area, wavelength, speed, activity, and swim speed) and six conditions (GWS, TS, MS, SR, MR, and NGM). Categorical changes were encoded numerically (increase = 1, no change = 0, decrease = −1) and visualized as a categorical map using the seaborn and matplotlib libraries in Python. Each cell represents the directional change in a given metric under a specific condition, with green indicating an increase, red indicating a decrease, and gray indicating no change. Statistical analysis provided by GraphPad 11.&nbsp; Vertical dividers were used to delineate condition groups.
